# Vaccination with inactivated SARS-CoV-2 vaccine TURKOVAC induces durable humoral and cellular immune responses up to 8 months

**DOI:** 10.3389/fmed.2025.1524393

**Published:** 2025-04-28

**Authors:** Seçil Yılmaz, Ahmet Eken, Zafer Sezer, Burcu Şen Bağcı, Serife Erdem, Medine Doğan Sarıkaya, Busra Kaplan, Ahmet Inal, Adnan Bayram, Gamze Kalın Unuvar, Gokmen Zararsız, Serra İlayda Yerlitas, Nuri Cakir, Shaikh Terkis Islam Pavel, Muhammet Ali Uygut, Hazel Yetiskin, Ates Kara, Aykut Ozdarendeli

**Affiliations:** ^1^Genome and Stem Cell Center, Erciyes University, Kayseri, Türkiye; ^2^Department of Medical Biology, Faculty of Medicine, Erciyes University, Kayseri, Türkiye; ^3^Department of Medical Pharmacology, Faculty of Medicine, Erciyes University, Kayseri, Türkiye; ^4^Good Clinical Practise Centre (IKUM), Erciyes University, Kayseri, Türkiye; ^5^Vaccine Research, Development and Application Centre (ERAGEM), Erciyes University, Kayseri, Türkiye; ^6^Department of Medical Microbiology, Faculty of Medicine, Erciyes University, Kayseri, Türkiye; ^7^Department of Anesthesiology and Reanimation, Faculty of Medicine, Erciyes University, Kayseri, Türkiye; ^8^Infectious Diseases Clinic, Department of Infectious Diseases, Faculty of Medicine, Erciyes University, Kayseri, Türkiye; ^9^Department of Biostatistics, Faculty of Medicine, Erciyes University, Kayseri, Türkiye; ^10^Pediatric Infectious Department, Faculty of Medicine, Hacettepe University Hospitals, Ankara, Türkiye

**Keywords:** TURKOVAC, SARS-CoV-2, vaccine, memory response, humoral, cellular

## Abstract

**Background:**

The rapid spread of the SARS-CoV-2 virus has led to a global health crisis, necessitating swift responses in medical science, mainly through vaccination strategies. While short-term vaccine effectiveness is evident, immune protection’s long-term effects and duration remain incompletely understood. Systematic monitoring of these responses is essential for optimizing vaccination strategies.

**Aims:**

This study aimed to explore the durability of antigen-specific T and B cell responses and antibody levels up to 8 months post-immunization with the inactivated TURKOVAC vaccine in volunteers. Additionally, the impact of two versus three doses of vaccination on these parameters was analyzed.

**Methods:**

Volunteers (*n* = 80) received two or three doses of TURKOVAC. Spike-specific B cells, CD4^+^ T cells, CD8^+^ T cells, and antibody levels were measured at multiple time points post-immunization.

**Results:**

Spike-specific B cells remained elevated up to 8 months post-immunization. SARS-CoV-2-specific CD4^+^ and CD8^+^ T cells peaked at 4 months but declined thereafter. TURKOVAC resulted in durable antigen-specific humoral and cellular immune memory with distinct kinetics. Still, most assessments observed no significant differences between two and three doses, except for antigen specific-IL-2 and CD4^+^ LAMP1 responses.

**Conclusion:**

TURKOVAC vaccination induces durable immune responses, with spike-specific B cells persisting up to 8 months and T cell responses peaking at 4 months before declining. These findings suggest that TURKOVAC contributes to long-term immune protection against SARS-CoV-2.

## 1 Introduction

In the Chinese city of Wuhan at the end of 2019, a brand-new coronavirus arose and spread an uncommon viral pneumonia outbreak (1). Due to the new virus’s high degree of sequence homology (79.6%) with the original SARS-CoV, it has been given the name “severe acute respiratory syndrome coronavirus 2” (SARS-CoV-2) (2). The SARS-CoV-2 is an enveloped, positive-sense, single-stranded RNA virus which belongs to the betacoronaviridae family (3, 4) Spike (S), nucleocapsid (N), envelope (E), and membrane (M) are the four structural proteins of SARS-CoV-2, with S and N viral proteins being the most immunogenic (5). This new coronavirus disease, also known as COVID-19 or coronavirus disease 2019, has spread quickly over the world due to its high transmission potential (1).

The COVID-19 pandemic and the resulting rise in deaths have made it crucial to create powerful SARS-CoV-2 vaccines worldwide, and this endeavor has been given top priority (6). SARS-CoV-2 vaccines are being evaluated using various vaccine platforms at preclinical and clinical levels, including nucleic acid (e.g., mRNA-1273, BNT162b2), viral vector (e.g., AZD1222/ChAdOx1, Gam-COVID-Vac-rAd26/rAd5), protein subunits (e.g., ZF2001, NVX-CoV2373), inactivated virus (e.g., CoronaVac, TURKOVAC) among others (7).

Following COVID-19 vaccination, effective immune responses necessitate a sequence of biological interactions between dendritic cells and T cells, B cells and T cells (8, 9). Virus-specific CD4^+^ T cells can develop into T follicular helper (Tfh) and Th1 cell subsets after immunization. The interaction of SARS-CoV-2-specific Tfh cells with B cells is essential for the development of neutralizing antibody responses and sustained humoral immunity. Tfh cells operate as “help” signals for B cells (9, 10).

Inactivated vaccines use the whole virus instead of specific parts of it as an immunogen. This method may generate a broader range of antibodies targeting various parts of the virus, known as epitopes, than vaccines that only use specific fragments of the virus (11).

TURKOVAC is an inactivated whole-virion vaccine developed from the SARS-CoV-2 strain hCoV-19/Turkey/ERAGEM-001/2020 isolated from a confirmed COVID-19 patient in Turkey (12). In preclinical trials, TURKOVAC was found to stimulate a strong immune response in BALB/c mice, thanks to its high immunogenicity (13). The vaccine’s efficacy in protecting against the lethal SARS-CoV-2 threat was shown in the K18-hACE2 animal model. Ferret models showed high viral clearance rates, similar to those observed during safety evaluations of the upper respiratory tract (13). Additionally, safety and immunogenicity analyses were performed on TURKOVAC (3 μg/0.5 mL and 6 μg/0.5 mL) in phase I and II trials. A phase I trial with a 21-day dosing schedule showed that 84% of vaccinated subjects displayed neutralizing antibodies that were indistinguishable between the two vaccine doses administered. Antibodies specific to anti-SARS-CoV-2 were found in the serum of all 43 volunteers treated with the vaccine on the 43rd day. A phase II trial conducted with a 28-day dosing schedule showed that the total immunoglobulin (Ig)G response against SARS-CoV-2 as measured by ELISA was significantly higher in the 6 μg group compared to the 3 μg group, but there was no significant difference in neutralizing antibody titers or T cell response as determined by ELISPOT (14).

Although TURKOVAC has completed phase 3 clinical trials, it has only received emergency authorization in Turkey (13). Given the continued need for data on inactivated vaccines, assessing the durability of immune responses to TURKOVAC remains essential.

Previous studies have evaluated the immune response following a homologous third dose of TURKOVAC, particularly with a 32-week post-booster follow-up (15). However, these studies did not directly compare the immune responses elicited by the second and third doses. Moreover, they lacked detailed assessments of antigen-specific B cells, Tfh cells, and memory T cell kinetics across multiple time points.

In this study, serum and peripheral blood samples were collected from 80 volunteers without prior suspicion or diagnosis of COVID-19 at six time points before and following vaccination. This allowed us to investigate the sustainability of virus-specific immunity induced by TURKOVAC and to compare immune responses between second- and third-dose recipients directly. Unlike previous studies, we provide a more detailed analysis of the kinetics of vaccine-induced humoral and cellular immunity responses, including antigen-specific B cells, CD4+ T cells, and CD8+ T cells, along with their memory subpopulations at six distinct time points.

## 2 Materials and methods

### 2.1 Formulation TURKOVAC

TURKOVAC was produced using the SARS-CoV-2 strain (hCoV-19/Turkiye/ERAGEM-001/2020; GenBank MT327745.1, GISAID EPI_ISL_424366) isolated from a patient at Kayseri City Training and Research Hospital, Türkiye (12). The virus was propagated in Vero cells for 72–96 h at an MOI of 0.05. The infected cell supernatant was harvested and inactivated with beta-propiolactone (1:4,000 v/v at 2–8°C for 6 h). After clarification and ultrafiltration, a second inactivation was performed with beta-propiolactone (1:2,000 v/v at 2–8°C for 6 h). The purified virus was then adsorbed onto an aluminum hydroxide adjuvant (alhydrogel) to formulate the vaccine (13). The vaccine was a liquid formulation containing 3 μg of total protein with 0.5 mg aluminum hydroxide adjuvant in 0.5 mL PBS, and was preservative-free.

### 2.2 Study design and clinical sample collection

In this study, the humoral and cellular immune responses in the peripheral blood of 80 volunteers following the TURKOVAC vaccine were studied. Eighty volunteers who were scheduled to receive the TURKOVAC vaccine were included in the study after obtaining approval from Erciyes University Clinical Research Ethics Committee (2021/74, 1 February 2021; 17 June 2021, 2021/396) and the Turkish Ministry of Health (9 February 2021; E-24931227-514.02.01-339886; 18 June 2021; E-66175679-514.02.01-463635). Two separate approvals were required as an additional protocol was introduced during Phase 2 to assess the need for a third dose.

The study was conducted in accordance with the Declaration of Helsinki, national regulations, and institutional guidelines and is registered at ClinicalTrials.gov (NCT04824391).

All clinical specimens were collected after informed written consent was obtained. All participants provided written informed consent before undergoing pre-study examination by signing the Informed Consent Form. Inclusion criteria for volunteers were the absence of suspected or diagnosed COVID-19. Exclusion criteria were defined as the absence of chronic diseases.

Among the 37 volunteers who received two doses, ages ranged from 18 to 53 years with a Body Mass Index (BMI) range of 18.8 to 32.0 (9 females, 28 males). Among the 43 volunteers who received three doses, ages ranged from 20 to 57 years with a BMI range of 18.3 to 32.0 (11 females, 32 males).

**Figure 1:**
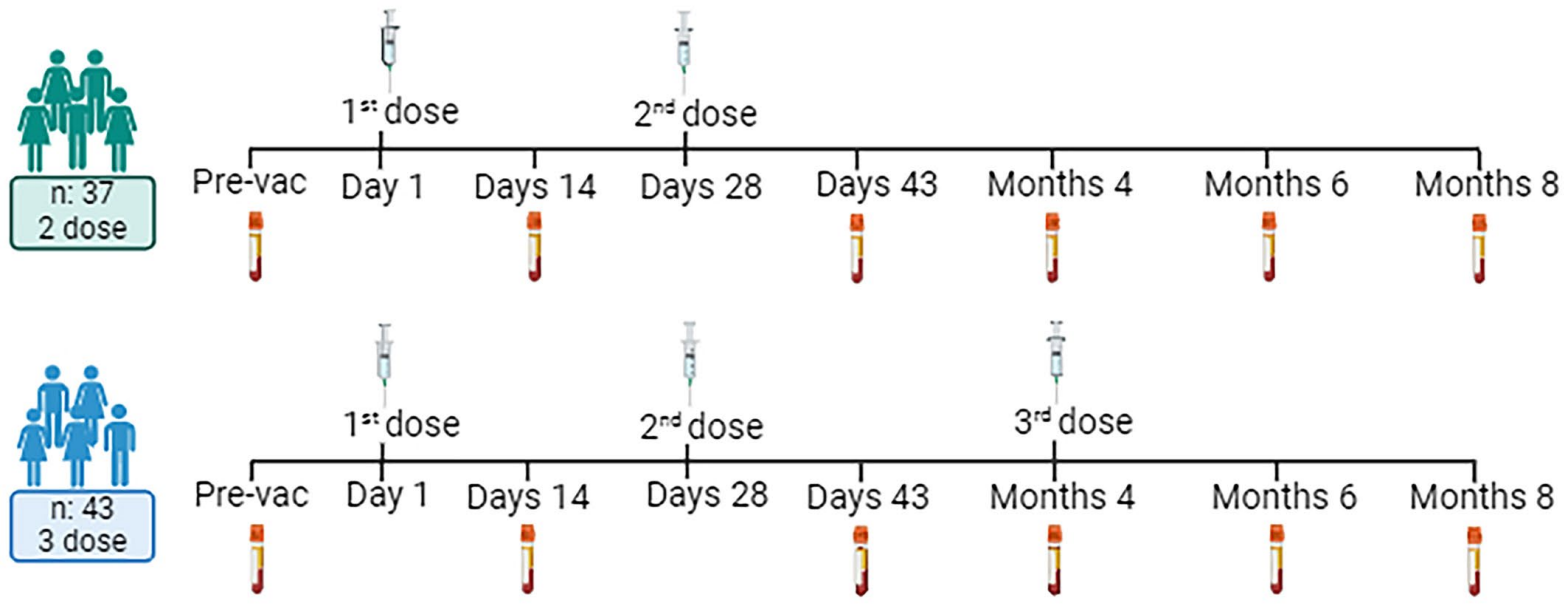
Experimental design and volunteer demographic information. Created with BioRender.com.

Peripheral blood samples were collected at six time points: day (D) 0 (pre-vaccination), D14, D43 (2 weeks after the second immunization), month (M) 4 (before the third immunization), M6, and M8 (Figure 1) (15).

### 2.3 Immunization schedule and sample selection and sample processing

Thirty-seven volunteers received two doses of immunization (Day 0 and 28), and 43 volunteers received 3 doses of immunizations (Day 0, 28, and 4 months). Peripheral blood samples collected 10 mL per patient into hemogram tubes containing EDTA were processed using Ficoll Paque for peripheral mononuclear cell isolation. Briefly, whole blood was diluted with a 1:1 Hank’s balanced salt solution was overlayed onto 15 mL of Ficoll Paque. The PBMC was collected from the interface after centrifugation at 400 × G for 45 min. PBMCs were counted with trypan blue were stored at -liquid nitrogen until use.

### 2.4 Detection of spike-specific memory B cells

For the detection of SARS-CoV-2-specific memory B cells, cryopreserved PBMCs were thawed in a 37°C water bath and washed with 5 mL of complete medium consisting of RPMI-1640 supplemented with 10% heat-inactivated FBS, 1% L-glutamine, and 1% Penicillin–Streptomycin. The PBMCs were then counted and evenly distributed in a 96-well U-bottom plate at a density of 5 × 10^5^ cells/well, which was blocked with Fc-block on ice for 10 min. Following this, the cells were stained with a protein mix containing 200 ng of biotinylated Spike protein (BioLegend, #793906) and 50 ng of BV421 Streptavidin (BioLegend, #405226) and incubated at 4°C for 1 h. After washing with Stain Buffer (PBS supplemented with 2% FBS), the cells were stained with an antibody cocktail consisting of CD19-FITC (BioLegend, #363008), CD3-APC (BioLegend, #300312), IgD-PE Dazzle (BioLegend, #348240), CD27-APC CY7 (BioLegend, #302816), CD38-Alexa 700 (BioLegend, #303524), CD20-BV510 (BioLegend, #302340), IgM-PerCP Cy5.5 (BioLegend, #314512), and IgG-PE Cy7 (BioLegend, #410722), used according to the manufacturer’s instructions without additional dilution (5 μL/test). Finally, the cells were washed twice with Stain Buffer and analyzed by flow cytometry using a BD FACS AriaIII. Dead cells were excluded from gating, and the live lymphocyte population was selected. Due to number of channels available in our flow cytometry, and their occupation with T and B cell markers, live dead stain could not be included. Nevertheless, cells were counted with trypan blue, and only live cells were included in the calculations, additionally gates in FSC/SSC plots were drawn to exclude debris and dead cells to the best of our ability. Cells were then gated on CD19+ cells for total B cell analysis (Supplementary Figure 1). Data analysis was performed using FlowJo Software version 10.0.7.

### 2.5 Detection of antigen specific activation of CD4^+^ and CD8^+^ T cells via AIM assay

In the activation-induced markers (AIM) assay, PBMCs were seeded at a density of 5 × 10^5^ cells/well in a 96-well U-bottom plate. Spike peptide library PepMix (Vial 1: 155 peptide, Vial 2: 156 peptide) (JPT Peptide Technologies, #PM-CVHSA-S) was reconstituted in 50 μL/vial DMSO and stock was prepared. 0.133 μL of stock PepMix was added to the cells per well and incubated at 37°C for a 24-h stimulation period. At the end of the stimulation, the cells were blocked at 4°C in the dark for 10 min using Fc-block (Biolegend, #422302) at a concentration indicated by the manufacturer. The cells were stained for 30 min in the dark and at 4°C using the following markers to determine antigen-specific activation of CD4^+^ and CD8^+^ T cells in 25 μL volume, 1.25 μL /well; CD3-APC (Biolegend, #300312), CD4-PE (Biolegend, #357404), CD8-FITC (Biolegend, #344704), CD137-BV421 (Biolegend, #309820), OX40-BV510 (Biolegend, #350026), LAMP-1-PE/Dazzle™ 594 (Biolegend, #328646), and CD69-Alexa Fluor 700 (Biolegend, #310922). For CD8^+^ T cell activation, antigen-specific responses were assessed based on LAMP-1 and CD137 expression. LAMP-1 expression was measured upon spike peptide challenge to determine CD8^+^ T cell activation. The detection strategy for CD137 expression followed the same gating and analysis approach. For Tfh cells, the following mix was used for staining: CD3-APC, CD4-PE, CXCR5-PB (Biolegend, #356918), OX40-BV510, CD69-Alexa 700, CD45RO-FITC (Biolegend, #304242), CCR6-PerCP/Cy5.5 (Biolegend, #353406), CXCR3-PE/Dazzle™ 594 (Biolegend, #353736), and CD40L-PE (Biolegend, #310832). The staining was performed in 25 μL volume, 1.25 μL /well dilution for each antibody. The cells were washed with Staining Buffer and examined in flow cytometry (BD FACS AriaIII). Data were analyzed with FlowJo Software version 10.0.7.

### 2.6 Detection of IL-2 cytokine level

The PBMCs were seeded at a density of 5 × 10^5^ cells/well into 96-well round-bottom plates in 100 μL/well of complete medium RPMI. For IL-2 detection, supernatants were collected after 3 days of antigen-specific T cell stimulation. The quantification was performed using a standard curve-based approach as per the manufacturer’s instructions.

The PBMCs were distributed into 96-well round-bottom plates in 100 μL/well of complete medium RPMI medium. The PepMix^™^ SARS-CoV-2 (Spike Glycoprotein) (JPT Peptide Technologies, #PM-CVHSA-S) vials (Vial 1: 155 peptide, Vial 2: 156 peptide) were added into the plate as 0.133 μL/well of peptide mix from 50 μL stock. Antigen-specific T cell stimulation was continued for 3 days at 37°C, and the supernatants were collected. For IL-2 detection, supernatants were collected after 3 days of antigen-specific T cell stimulation. The quantification was performed using a standard curve-based approach as per the manufacturer’s instructions. Detection and measurement of cytokine were performed using a LEGEND MAX^™^ Human IL-2 ELISA Kit (BioLegend, #431807) according to the manufacturer’s instructions.

### 2.7 Measurement of spike-specific plasma antibody isotype levels

The anti-SARS-CoV-2 IgG ELISA test kit (Euroimmun, #EI-2606-9601-10-G) was used to detect antibodies in the plasma samples. All chemicals, solutions, and materials, including 96-well microplates, were initially brought to room temperature. Diluted samples at 1:100 and 1:500 were added to the wells in a volume of 100 μL. After incubation at 37°C for 60 min, the wells were washed three times with 200 μL of wash solution.

Subsequently, 100 μL of enzyme-conjugated anti-human IgG1-HRP, anti-human IgG2-HRP, anti-human IgG3-HRP, and anti-human IgG4-HRP (all from Southern Biotech) was added, and the plates were incubated in the dark at 37°C for 30 min. Following incubation, the wells were washed three times with 200 μL of wash solution.

Then, 100 μL of chromogen/substrate solution was added to each well and incubated in the dark at 37°C for 30 min. To stop the reaction, 100 μL of stop solution was added to each well, and the absorbance was measured at 450 nm using an ELISA reader.

### 2.8 Statistical analyses

Histogram, q-q plots, and Shapiro–Wilk’s test were applied to assess the data normality. Levene test was used to test variance homogeneity. For B cells, T cells, and Tfh cells, a shifted logarithmic transformation (log10(x + 1)) was applied to account for highly skewed distribution. Data are summarized using the arithmetic mean and standard deviation for B cells, T cells, and Tfh cells. Since the data did not follow normal distribution after logarithmic transformation for cytokines, analyzes were continued with nonparametric methods. Cytokine data are summarized using the median and interquartile range. Between-group comparisons (*p*^†^) were performed using a two-sided independent samples t-test and Mann–Whitney U test. Within-group comparisons (*p*^‡^) were performed using linear mixed-effect models and Friedman tests. For multiple comparisons, Bonferroni and Nemenyi tests were applied simultaneously. All baseline values were one (1.00) before transformation. The standard deviation statistic was not calculated, and this time point was excluded from within-group comparisons. Since shifted logarithmic transformation (log(x + 1)) was applied, this quantity, as well as the mean statistic, is computed as 0.30. A *p*-value of <0.05 was considered statistically significant. All analyses were conducted using TURCOSA (Turcosa Analytics Ltd. Co., Turkey)^1^ and R 4.2.0^2^ software.

## 3 Results

### 3.1 TURKOVAC vaccination induces spike-specific IgM^+^ and IgG^+^ memory B cells detectable up to 8-month post-vaccination

**Figure 2:**
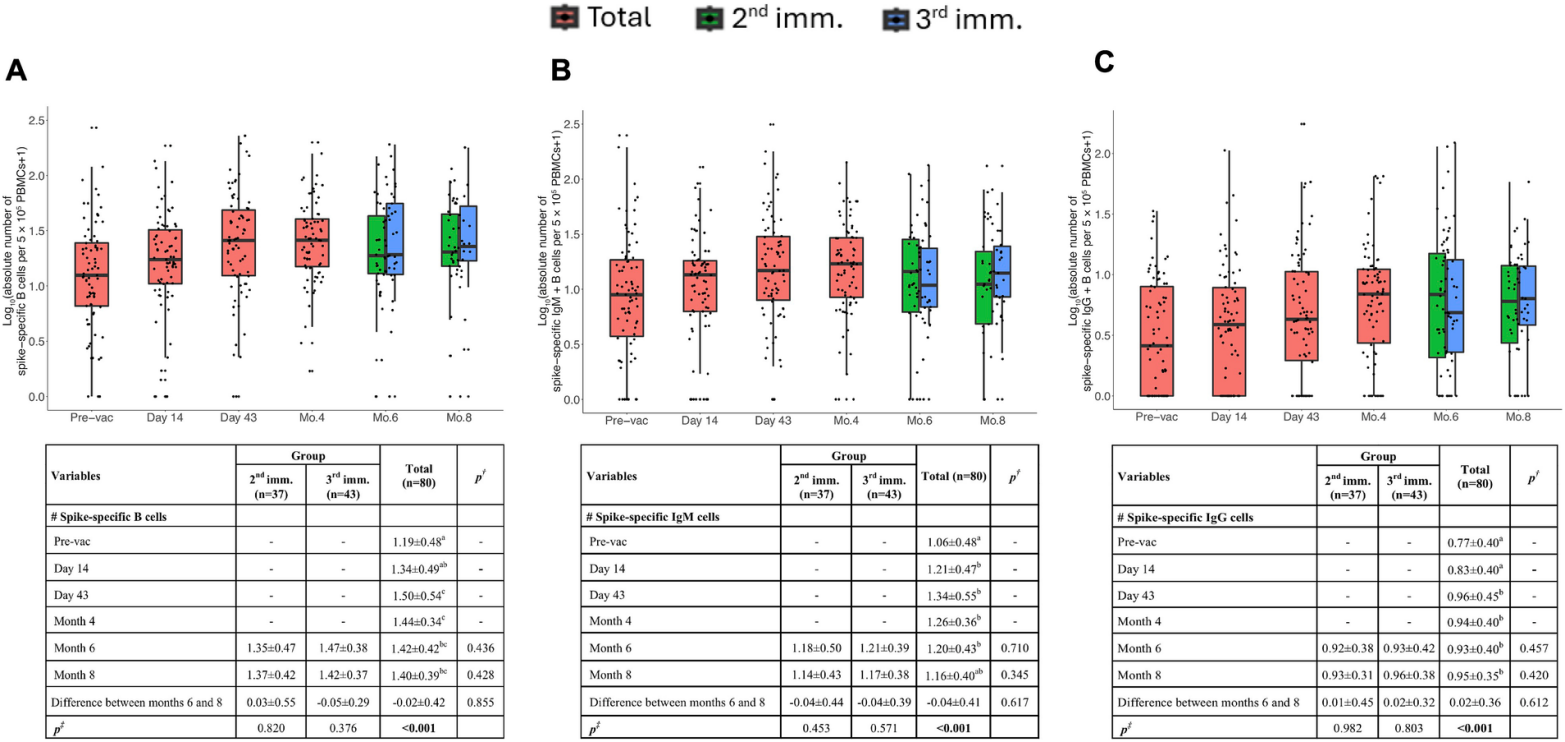
Memory B cell responses induced by TURKOVAC vaccination. The absolute number of spike-specific memory B cells **(A)**, IgM+ **(B)**, and IgG+ **(C)** B cells per 5 × 10^5^ PBMCs before and after vaccinations. Red, green, and blue indicate all volunteers, volunteers with two doses of vaccination, and volunteers with three doses of vaccination, respectively. Values are expressed as arithmetic mean and standard deviation; *p*^†^ indicates the significance for between-group comparisons; *p*^‡^ indicates the significance for within-group comparisons. All baseline values were one (1.00) before the transformation. Standard deviation statistic was not calculated, thus this time point was not included in within-group comparisons. Since shifted logarithmic transformation (log(x + 1)) was applied, this quantity, as well as the mean statistic, is computed as 0.30. Significant *p* values are shown in bold.

Spike-specific B cells exhibited a significant increase on the 43rd day and maintained this significance up to the 8th month (Figure 2A). The third dose did not exhibit a significant increase in antigen-specific B cells compared to the second dose. This outcome might have been influenced by potential freezing and thawing effects on sample processing.

Additionally, we examined the isotype of Ig immunoglobulins. Antigen specific B cells were observed after the first immunization, on the 14th day and remained significant up to the 8th month. Spike-specific IgM^+^ or IgG^+^ B cells were detectable after the second immunization on day 43 (Figures 2B,C). Notably, flow cytometric assessment was not sensitive enough to detect the increase between the second and third doses. Altogether, these data argue that the vaccination with TURKOVAC effectively led to a detectable presence of IgG^+^ IgM^+^ Spike protein-specific B cells.

### 3.2 Spike-specific serum IgG titers decrease to basal levels at 8 months after TURKOVAC vaccination

**Figure 3:**
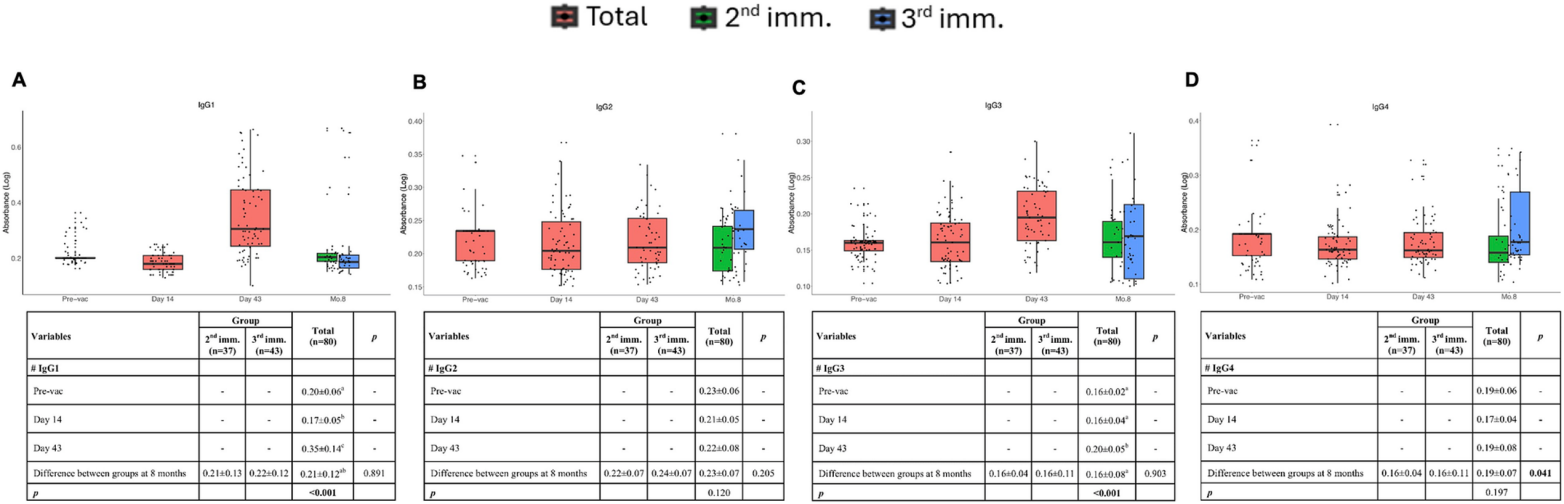
IgG subclasses at different time points after vaccinations. Absorbance values of IgG1 **(A)**, IgG2 **(B)**, IgG3 **(C)**, and IgG4 **(D)** before vaccination and after the first, second, and third doses of vaccination. Red, green, and blue indicate all volunteers, volunteers with two doses of vaccination, and volunteers with three doses of vaccination, respectively. Values are expressed as arithmetic mean and standard deviation; *p*^†^ indicates the significance for between-group comparisons; *p*^‡^ indicates the significance for within-group comparisons. All baseline values were one (1.00) before the transformation. Standard deviation statistic was not calculated, thus this time point was not included in within-group comparisons. Since shifted logarithmic transformation (log(x + 1)) was applied, this quantity, as well as the mean statistic, is computed as 0.30. Significant *p* values are shown in bold.

To assess the long-term durability of the IgG response, levels of the IgG subclasses were measured using ELISA. In the previous phase I and II study, it was reported that Spike-specific total IgG levels increased after the second immunization (14). In the present study, we aimed to examine IgG subsets in more detail (Figures 3A,C). Spike-specific IgG1 and IgG3 were induced after the second dose of immunizations. At 8 months the levels of IgG1 returned to the baseline levels and IgG3 also dropped significantly, consistent with the reports with other COVID vaccines, highlighting the potential need for booster vaccinations (Figures 3A,C).

There was no induction of spike-specific IgG2 following three doses of immunization. However, after the third dose of immunization induction of some spike-specific IgG2 and IgG4 was observed (Figures 3B,D). These findings indicate that TURKOVAC induces spike-specific IgG1 and IgG3 production following two doses and suggest the necessity of booster doses by 8 months.

### 3.3 TURKOVAC vaccination induces spike protein specific CD4^+^ and CD8^+^ memory T cells following two doses of immunization detectable up to at 8 months post-vaccination

**Figure 4:**
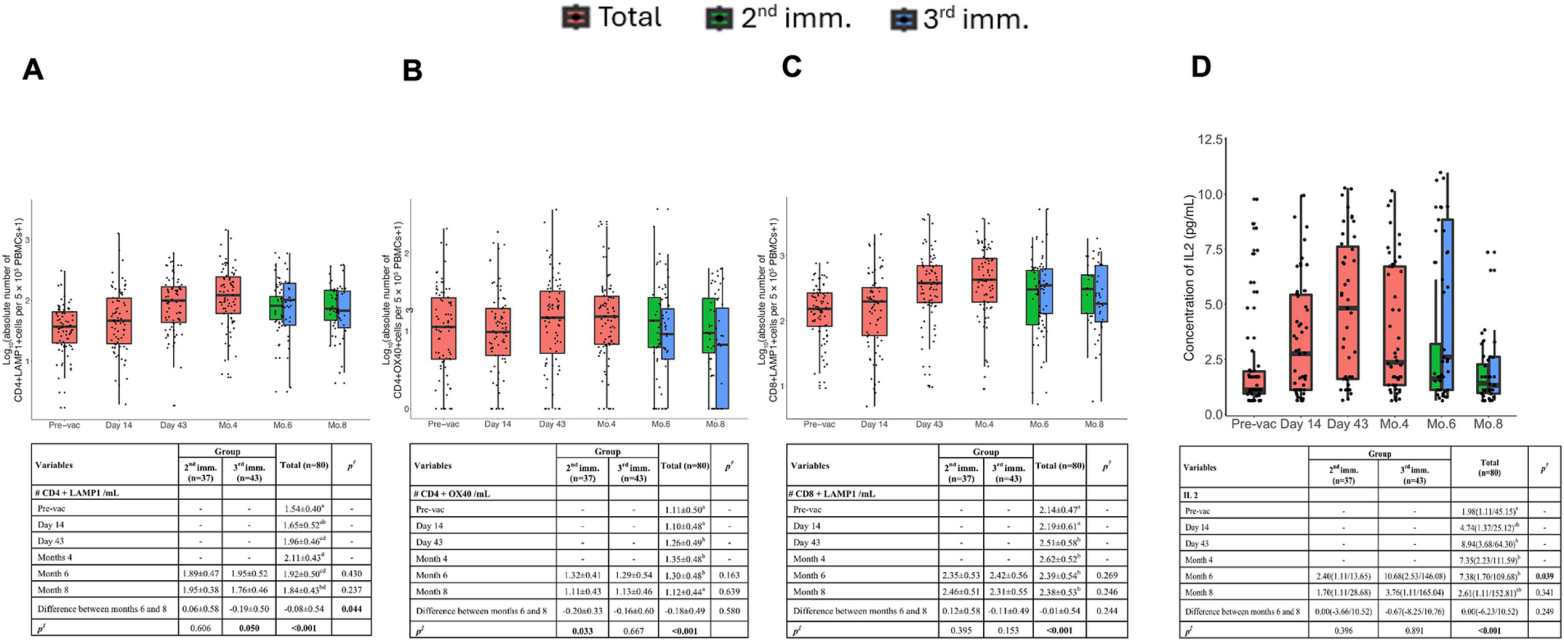
Memory T cells and IL-2 cytokine concentration at six time points post-vaccination after stimulation with SARS-CoV-2 Spike peptides. The absolute number of antigen-specific LAMP-1+ **(A)** and OX40+ **(B)** T cells among CD4+ T cells per 5 × 10^5^ PBMCs over time. **(C)** Absolute number of antigen-specific LAMP-1+ T cells among CD8+ T cells per 5 × 10^5^ PBMCs over time. **(D)** Concentration of IL-2 cytokine before vaccination and after the first, second, and third doses of vaccination. Red, green, and blue indicate all volunteers, volunteers with two doses of vaccination, and volunteers with three doses of vaccination, respectively. Values are expressed as arithmetic mean and standard deviation; *p*^†^ indicates the significance for between-group comparisons; *p*^‡^ indicates the significance for within-group comparisons. All baseline values were one (1.00) before the transformation. Standard deviation statistic was not calculated, thus this time point was not included in within-group comparisons. Since shifted logarithmic transformation (log(x + 1)) was applied, this quantity, as well as the mean statistic, is computed as 0.30. Significant *p* values are shown in bold.

Activation-induced markers assay method was adapted from prior studies was used to determine antigen-specific memory T cell responses (16). LAMP1^+^ CD4^+^ T cells remained detectable, even 8 months after immunization (Figure 4A). In addition, a significant increase in LAMP1^+^ CD4^+^ T cells was observed after the third vaccination. Furthermore, OX40 expressing clones became detectable after the second vaccination, and these cells persisted until the 6th month (Figure 4B). Unlike LAMP1, no significant increase in OX40 expression was detected following the third dose compared to the second dose.

Antigen-specific activation of CD8^+^ memory T cells was assessed by LAMP1 and CD137 expression. LAMP-1 was significantly upregulated upon Spike peptide challenge in the samples of volunteers on day 43 and remained detectable until the 8th month (Figure 4C). However, CD137 expression did not show significant upregulation upon antigenic stimulation.

Antigen-specific IL-2 production remained high up to 4 months post-vaccination but declined by the 6th month. Volunteers who received three doses exhibited significantly higher antigen-specific IL-2 production, suggesting that the third immunization enhanced IL-2-mediated T cell responses (Figure 4D).

### 3.4 TURKOVAC vaccination induces antigen specific memory Tfh cell subsets reactive against spike peptide pool detectable up to 4 to 6 months post-vaccination

**Figure 5:**
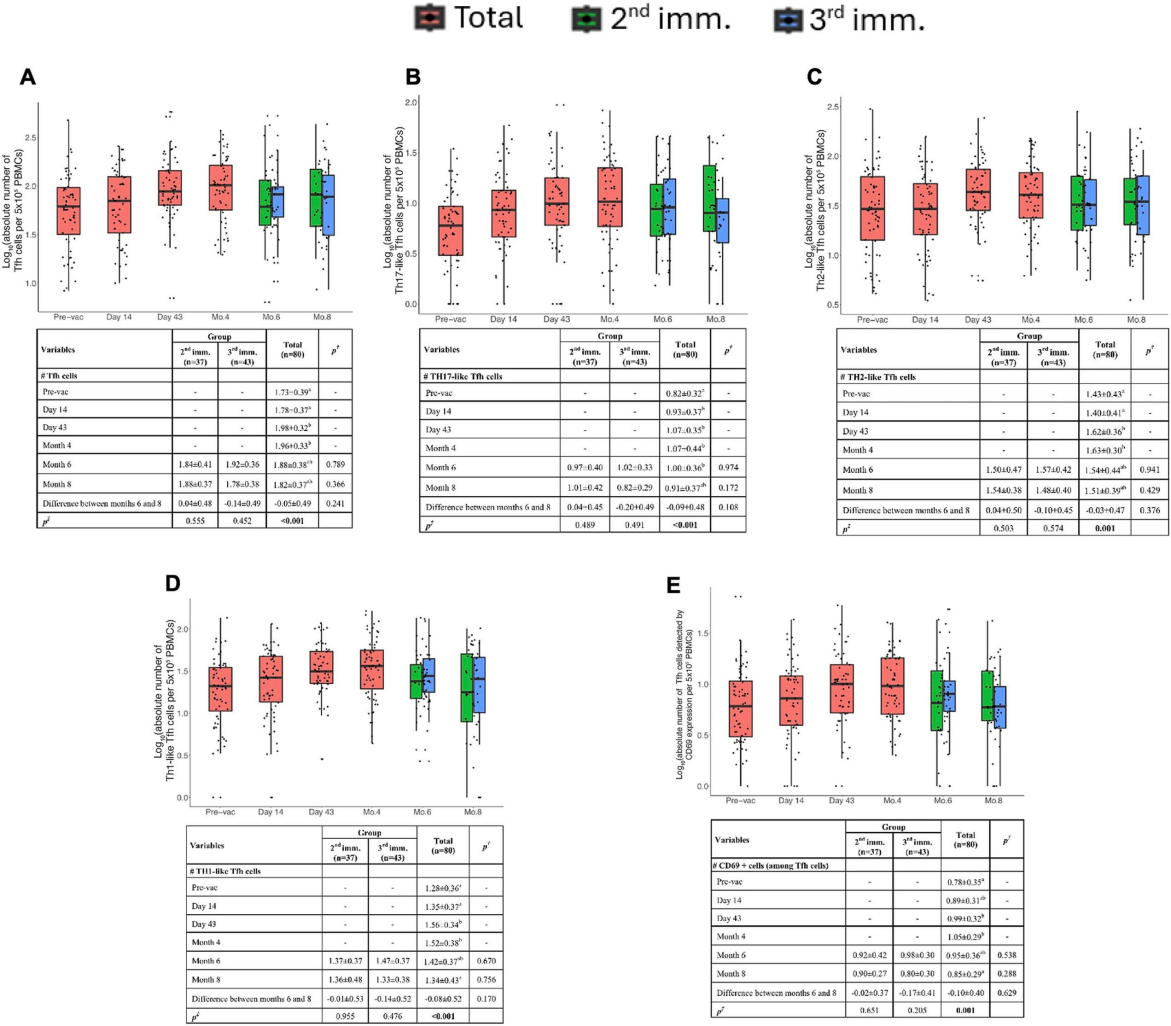
Spike-specific follicular helper T cell (Tfh) responses. **(A)** Absolute number of total Tfh cells (CD3^+^CD4^+^CD45RO^+^CXCR5^+^) per 5 × 10^5^ PBMCs. **(B)** Absolute number of Th17-like (CXCR3^−^CCR6^+^) Tfh cells per 5 × 10^5^ PBMCs. **(C)** Absolute number of Th2-like (CXCR3^−^CCR6^−^) Tfh cells per 5 × 10^5^ PBMCs. **(D)** Absolute number of Th1-like (CXCR3^+^CCR6^−^) Tfh cells per 5 × 10^5^ PBMCs. **(E)** Absolute number of activated SARS-CoV-2 Spike-specific Tfh cells detected by CD69 expression per 5 × 10^5^ PBMCs. Red, green, and blue indicate total volunteer values, volunteer values with two doses of vaccination, and volunteer values with three doses of vaccination, respectively. Values are expressed as arithmetic mean and standard deviation; *p*^†^ indicates the significance for between-group comparisons; *p*^‡^ indicates the significance for within-group comparisons. All baseline values were one (1.00) before the transformation. Standard deviation statistic was not calculated, thus this time point was not included in within-group comparisons. Since shifted logarithmic transformation (log(x + 1)) was applied, this quantity, as well as the mean statistic, is computed as 0.30. Significant *p* values are shown in bold.

Total Tfh cell counts increased significantly after the second immunization (day 43), and these antigen-specific Tfh cells were detectable for up to 4 months post-immunization (Figure 5A). Tfh responses did not differ between 2nd and 3rd dose recipient donors concerning parameters tested.

Tfh cells can be grouped into subtypes such as Th1, Th2, and Th17-like cells. The data in the volunteers’ PBMCs showed the presence of Th17-like Tfh cells. These cells increased on the 14th day after the first vaccination and persisted up to the 8th month (Figure 5B).

Th1-like Tfh cells peaked at day 43 and began to decline after the 4th month (Figure 5D). Th2-like Tfh cells exhibited significant increases at both day 43 and 4 months post-vaccination (Figure 5C).

Similarly, Th1-like Tfh cells were significantly higher in volunteers after the second immunization. CD69 expression analysis indicated that antigen-specific Tfh cells were elevated at day 43 and persisted up to 4 months post-vaccination (Figure 5E).

Altogether, these findings suggest that antigen-specific memory Tfh cells were induced following two doses of TURKOVAC and were detectable up to 4 months post-vaccination.

## 4 Discussion

In the current study, spike-specific B and T cell immune responses of volunteers enrolled in a Phase II trial vaccinated with TURKOVAC, an inactivated vaccine against SARS-CoV-2, were investigated longitudinally up to 8 months post-immunization. The data presented herein showed that TURKOVAC vaccination is capable of generating spike-specific B cell clones with surface IgG and IgM expression, which were detectable as late as 8 months post-vaccination. Additionally, spike-specific serum IgG1 and IgG3 subsets declined at 8 months post-vaccination, while both CD4^+^ and CD8^+^ clones reactive to spike protein-derived peptides remained detectable at 8 months post-vaccination. Notably, no significant differences were observed between two and three doses in most immune parameters analyzed, except for IL-2 production and CD4^+^ LAMP1 expression, which were significantly higher in the three-dose group. The data presented herein support that TURKOVAC can mount protective cellular and humoral immunity against SARS-CoV-2.

Our data demonstrated a notable surge in spike-specific B cells by day 43 post-vaccination with TURKOVAC, which was durable up to 8 months post-vaccination. Additionally, both spike-specific IgG^+^ and IgM^+^ B cell clones were significantly elevated after two doses of TURKOVAC vaccination, and IgG^+^ clones remained detectable above pre-vaccination levels at 8 months post-vaccination. These results suggest that TURKOVAC is able to induce durable humoral immunity favoring class-switched IgG production. The booster dose did not significantly enhance B cell responses, suggesting that two doses were sufficient to generate a stable memory B cell pool. Studies with BioNTech revealed detectable IgG^+^ and IgM^+^ B cell clones as late as 6 months post-vaccination (17–19). Similarly, studies with CoronaVac revealed detectable RBD-specific total and IgG^+^ B cell clones 2 months after the third dose of vaccination (20). In a study conducted with Covaxin, antigen-specific B cells were detected for up to 6 months post-vaccination (21). Comparison of our results with those of BioNTech and other vaccine platforms indicates that immunization with TURKOVAC is successfully able to induce the expansion of spike-specific B cell clones and memory B cells, which may persist for up to 8 months post-vaccination.

Our study revealed that vaccination with two doses of TURKOVAC induced effective spike-specific plasma IgG1 and IgG3 levels, which were detectable 6 weeks post-immunization. IgG1 induction was more robust compared with IgG2, IgG3, and IgG4. By 8 months post-vaccination, antibody titers returned to baseline levels, indicating the potential need for booster immunizations at this time point. Two doses of immunization did not evoke IgG4, which has been reported to hinder the efficacy of immunizations.

In studies examining long-term plasma IgG kinetics, individuals who received CoronaVac, an inactivated COVID-19 vaccine, exhibited the highest levels of IgG1 and IgG3, with little IgG2 and IgG4 (22), similar to our results. Antigen-specific IgG1 and IgG3 levels of CoronaVac decreased approximately 3 months after the two-dose primary vaccination. Although IgG1 and IgG3 levels increased after a booster dose administered 1 year after primary vaccination, a decrease was observed after 3 months (22). In individuals who received mRNA vaccines, antigen-specific IgG1 levels returned to normal 6 months post-vaccination. These results demonstrate that TURKOVAC induces effective antigen-specific IgG1 and IgG3 responses against SARS-CoV-2, and that antibody levels return to normal by 8 months post-vaccination, comparable to the responses observed with currently available inactivated and mRNA-based SARS-CoV-2 vaccines (23).

Antigen-specific T cell responses were also assessed longitudinally following TURKOVAC vaccination. We employed AIM assays to detect both CD4^+^ and CD8^+^ T cells. In our study, CD4^+^LAMP1^+^ T cells and IL-2 production were significantly enhanced in the three-dose group, while other T cell parameters showed durable responses up to 8 months, regardless of the dosing regimen. This finding suggests that the third dose contributed to the enhancement of cellular immunity, particularly IL-2 signaling and CD4^+^ T cell activation.

Antigen specific T cell responses were also assessed longitudinally following vaccination of volunteers with TURKOVAC. We employed AIM assays to detect both CD4^+^ and CD8^+^ T cells. In our study, both CD4^+^LAMP1^+^ T cells and CD8^+^LAMP1^+^ T cells peaked at 4 months, and CD4^+^OX40^+^ T cells peaked at day 43, and all of them continued to exist until the 8th month.

Memory T cell responses following vaccination in healthy volunteers have been studied in other SARS-CoV-2 vaccines, with CD4^+^ and CD8^+^ T cell responses detected for up to 6 months after mRNA-1273, BNT162b2, Ad26.COV2.S, and NVX-CoV2373 vaccination (16, 17). BBV152/Covaxin induced a strong CD4^+^ T cell response in most individuals (85%), which was durable for 6 months (24). According to our results, IL-2 levels reached their highest level in the 6^th^ month with the effect of the 3^rd^ booster dose, and then a decrease was observed. IL-2 secreting multifunctional T cell profiles have also been seen in SARS-CoV-2 mRNA and inactive vaccines (24, 25). IL-2-secreting multifunctional T cell profiles began to decrease after day 43 after two doses of mRNA-1273 vaccination but remained present for up to 6 months (25). Also, after two doses of CoronaVac vaccination, the fraction of IL-2-producing CD4^+^ central memory T and CD4^+^ effector memory T cells increased from 1 to 6 months after the second dose and remained high throughout the follow-up period (26). In the case of inactivated virus vaccines, antigen-specific CD4^+^ T cell responses are commonplace, while the generation of CD8^+^ T cell responses is frequently not as anticipated (27). Our results show that TURKOVAC can produce antigen-specific both CD4^+^ and CD8^+^ T cells against SARS-CoV-2 which are detectable for up to 8 months. Studies conducted within 12 months after two doses of CoronaVac vaccination revealed that the fraction of RBD-specific CD4^+^ central memory T cells decreased at 6 months and preserved 12 months after the second vaccination, while RBD-specific CD8^+^ effector memory responses peaked at 3 months and then decreased over time (26). Six months after three doses of BBIBP-CorV, both CD4^+^ and CD8^+^ T cells were detectable, and CD8^+^ IFN-*γ*^+^ T cells expanded more than CD4^+^IFN-γ^+^ T cells (28). Collectively, our analyses of spike-specific memory T cells following immunizations with TURKOVAC revealed generation and maintenance of spike-specific CD4^+^ and CD8^+^ memory T cells up to 8 months.

Tfh cells play a crucial role in the development and maturation of humoral immune responses by providing costimulatory molecules and cytokines to B cells (29). Tfh cells are categorized into Tfh1 (CXCR3^+^CCR6^−^), Tfh2 (CXCR3^−^CCR6^−^), and Tfh17 (CXCR3^−^CCR6^+^) subgroups based on CCR6/CXCR3 expression (30). In our study, Th1-, Th2-, and Th17-like antigen-specific Tfh cells maintained their presence until the 6th month, and the dosing regimen did not significantly impact their persistence.

Overall, our findings indicate that TURKOVAC vaccination induces strong and durable humoral and cellular immune responses against SARS-CoV-2. While most immune parameters remained comparable between two- and three-dose regimens, IL-2 production and CD4^+^ LAMP1^+^ T cell activation were significantly enhanced by the third dose. This suggests that the third dose contributes to the enhancement of cellular immunity. Future studies comparing TURKOVAC with other inactivated COVID-19 vaccines in a side-by-side manner will be informative in determining its relative effectiveness.

## Data Availability

The original contributions presented in the study are included in the article/Supplementary material, further inquiries can be directed to the corresponding author.

## Footnotes

^1^ www.turcosa.com.tr

^2^ www.r-project.org
