# Supplementary material for: Vaccination with inactivated SARS-CoV-2 vaccine TURKOVAC induces durable humoral and cellular immune responses up to 8 months
Source: Front Med (Lausanne). 2025 Apr 28;12:1524393. doi: 10.3389/fmed.2025.1524393 (PMC12066321; doi:10.3389/fmed.2025.1524393)
Supplement: Supplementary file 1 [file Table_1.docx]

Supplementary Material

## Supplementary Figures


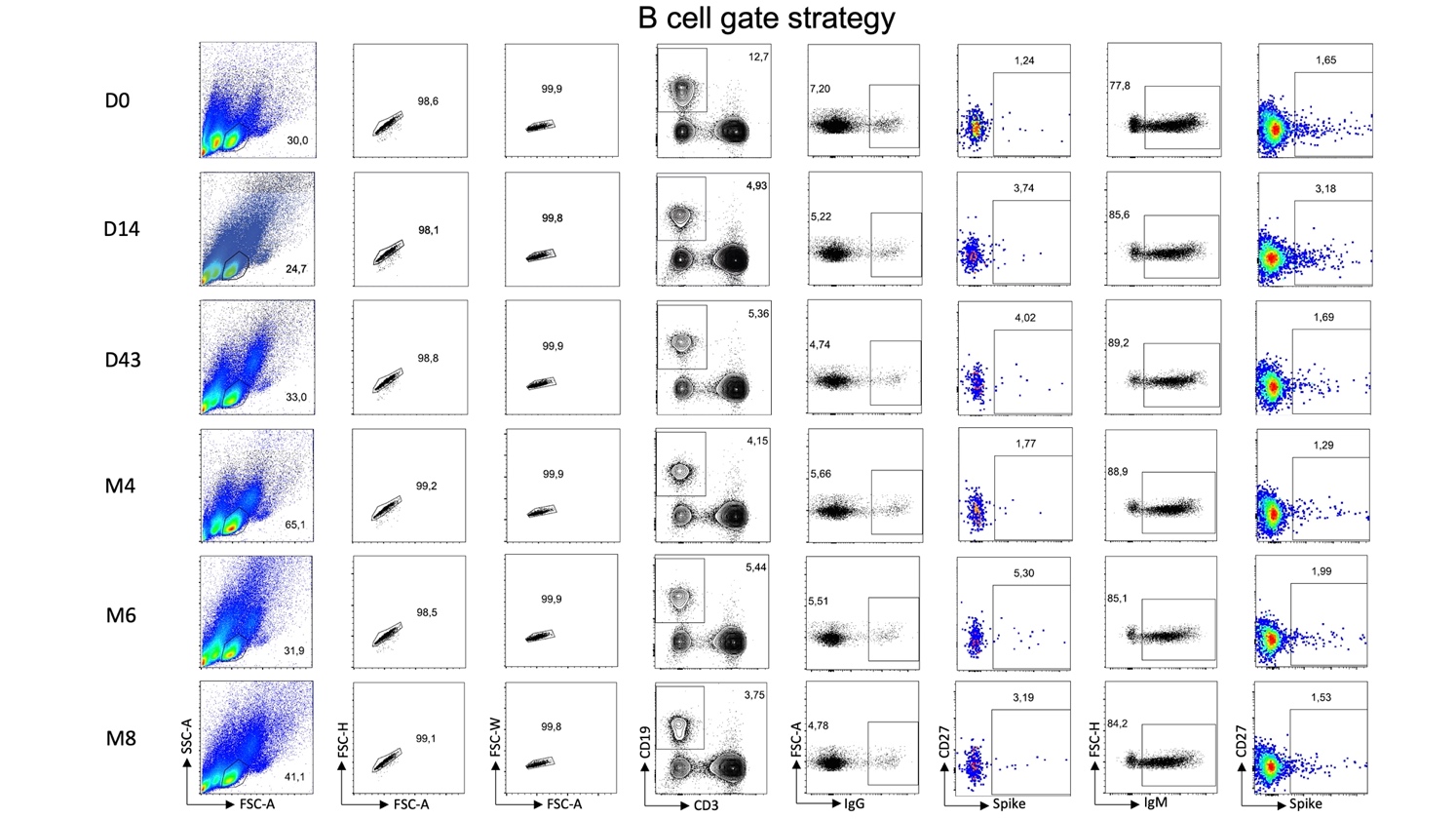


**Supplementary Figure 1.** Flow cytometry gating strategies for B cells. Cells were stained with antibodies against CD3, CD19, CD27, IgM, IgG. First, dead cells were excluded from gating and the live lymphocyte population was selected. Cells were then gated on CD19+ cells for analysis of total B cells. Spike+ IgG and Spike+ IgM cell populations were determined within this cell population.


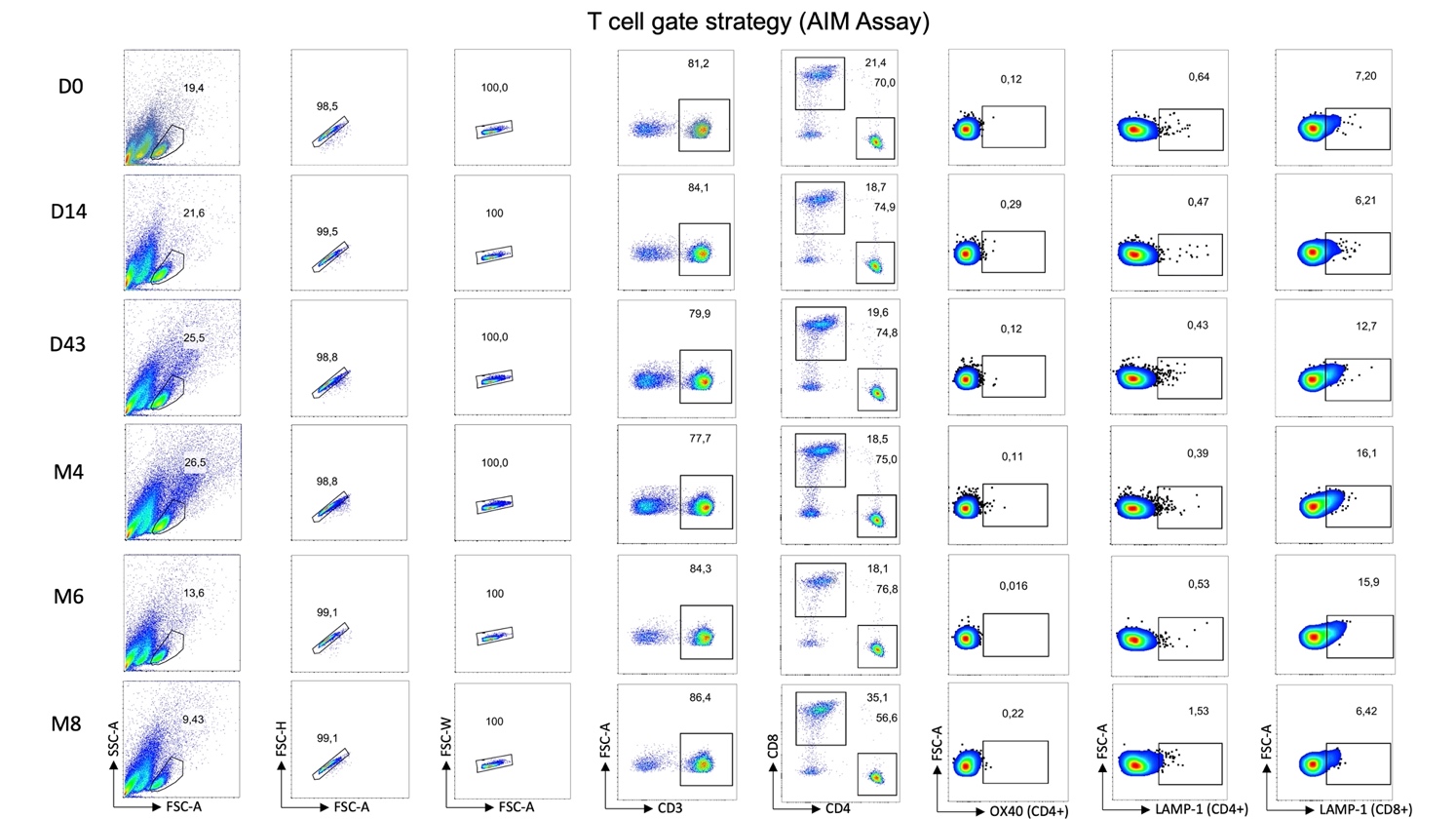


**Supplementary Figure 2.** Flow cytometry gating strategy of spike antigen-induced T cells. Dead cells were excluded from gating and the live lymphocyte population was selected. Cells were then gated on CD3+ cells for analysis of total T cells. CD8+ and CD4+ cell populations were determined within this cell population. OX40+ and LAMP-1+ cell populations were also identified within the CD4+ cell population. LAMP-1+ cell populations were identified among the CD8+ cell population.


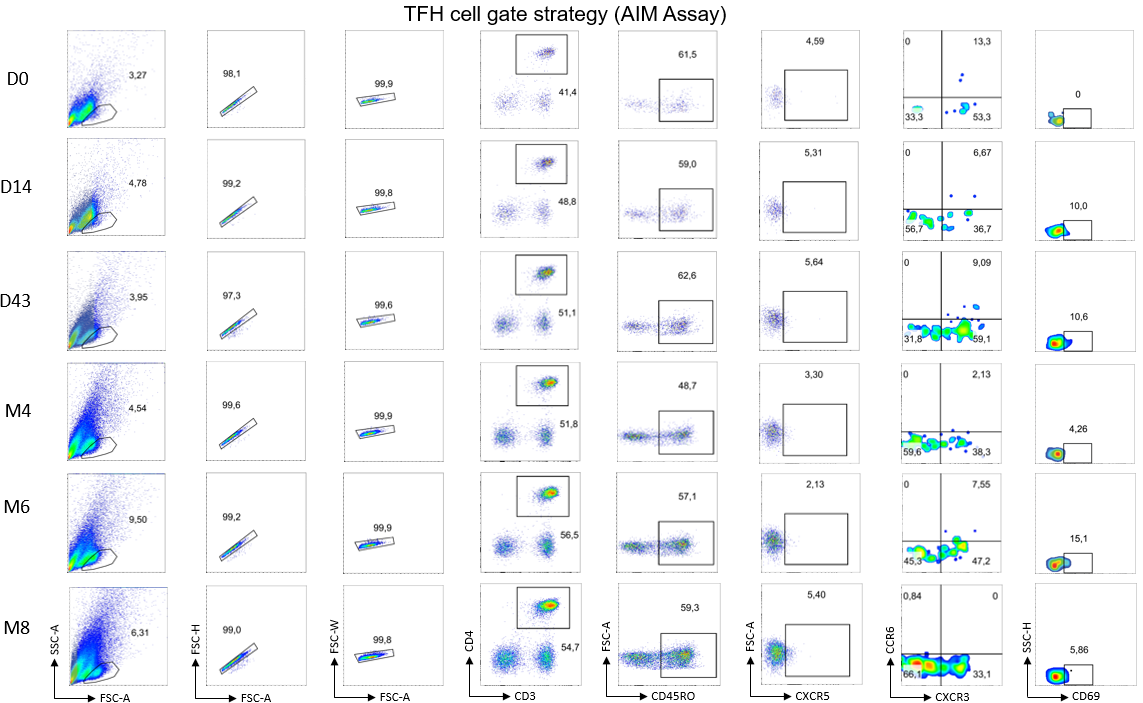


**Supplementary Figure 3.** Flow cytometry gating strategy of spike antigen-induced Tfh cells. Dead cells were excluded from gating and the live lymphocyte population was selected. CD3+CD4+ cells were then selected. CD45RO+ cell populations were identified within this cell population. CXCR5+ cell populations were also identified among the CD45RO+ cell population. Among the CXCR5+ cell population, CXCR3+CCR6+ (TH17-like Tfh), CXCR3+CCR6- (TH1-like Tfh), CXCR3-CCR6- (TH2-like Tfh) and CD69+ cell populations were quantified.
